# Comparative genomics among *Saccharomyces cerevisiae* × *Saccharomyces kudriavzevii* natural hybrid strains isolated from wine and beer reveals different origins

**DOI:** 10.1186/1471-2164-13-407

**Published:** 2012-08-20

**Authors:** David Peris, Christian A Lopes, Carmela Belloch, Amparo Querol, Eladio Barrio

**Affiliations:** 1 ‘Cavanilles’ Institute of Biodiversity and Evolutionary Biology, University of Valencia, Parc Científic, P.O. Box 22085, E-46071, Valencia, Spain; 2 Department of Biotechnology, Institute of Agrochemistry and Food Technology (CSIC), Valencia, Spain; 3 Yeast Biodiversity & Biotechnology Group. Instituto Multidisciplinario de Investigación y Desarrollo de la Patagonia Norte (IDEPA), CONICET, Universidad Nacional del Comahue, Neuquén, Argentina

## Abstract

**Background:**

Interspecific hybrids between *S. cerevisiae* × *S. kudriavzevii* have frequently been detected in wine and beer fermentations. Significant physiological differences among parental and hybrid strains under different stress conditions have been evidenced. In this study, we used comparative genome hybridization analysis to evaluate the genome composition of different *S. cerevisiae* × *S. kudriavzevii* natural hybrids isolated from wine and beer fermentations to infer their evolutionary origins and to figure out the potential role of common *S. kudriavzevii* gene fraction present in these hybrids.

**Results:**

Comparative genomic hybridization (CGH) and ploidy analyses carried out in this study confirmed the presence of individual and differential chromosomal composition patterns for most *S. cerevisiae* × *S. kudriavzevii* hybrids from beer and wine. All hybrids share a common set of depleted *S. cerevisiae* genes, which also are depleted or absent in the wine strains studied so far, and the presence a common set of *S. kudriavzevii* genes, which may be associated with their capability to grow at low temperatures. Finally, a maximum parsimony analysis of chromosomal rearrangement events, occurred in the hybrid genomes, indicated the presence of two main groups of wine hybrids and different divergent lineages of brewing strains.

**Conclusion:**

Our data suggest that wine and beer *S. cerevisiae* × *S. kudriavzevii* hybrids have been originated by different rare-mating events involving a diploid wine *S. cerevisiae* and a haploid or diploid European *S. kudriavzevii* strains. Hybrids maintain several *S. kudriavzevii* genes involved in cold adaptation as well as those related to *S. kudriavzevii* mitochondrial functions*.*

## Background

The development of molecular methods of yeast characterization has demonstrated that some wine and brewing *Saccharomyces* strains possess complex genomes composed by genetic elements from two or more species [1-7]. These strains are widely known as interspecific hybrids.

The best characterized industrial interspecific hybrid is the lager yeast *S. pastorianus*, originated from hybridization between *S. cerevisiae* and a *S. bayanus*-related yeast, which recently has been suggested to belong to the new species *S. eubayanus*[8]. The hybridization between *S. cerevisiae* and the cryotolerant *S. eubayanus* have been suggested as the result of selective pressures derived from brewing at low temperatures [8].

Other kind of natural *Saccharomyces* hybrids are those originated from hybridization between *S. cerevisiae* and *S. kudriavzevii*. These hybrids have mainly been isolated from wine and brewing environments [1-3].

The role of the *S. kudriavzevii* genome in these hybrids is unclear, since the known strains of this species have been found in decaying leaves from Japan and oak trees from Portugal and Spain [9,10], but not in fermentative industrial environments yet. The physiological evaluation of some of these *S. kudriavzevii* isolates showed that this species is characterized by a higher cryotolerance than *S. cerevisiae*, but a lower ethanol tolerance [11,12].

Albeit differences between *S. cerevisiae* × *S. eubayanus* and *S. cerevisiae* × *S. kudriavzevii* hybrids, the role of the *S. eubayanus* or *S. kudriavzevii* genomes in the hybrid seems to be similar, that is, the maintenance of good fermentative performance at low temperatures.

The characterization of a particular group of Swiss wine hybrids by PCR-RFLP, DNA arrays, ploidy analysis and gene dose determination by quantitative real-time PCR, evidenced the existence of a single common hybridization event to explain the origin of these hybrids followed by extensive chromosomal rearrangements including chromosome losses and the generation of chimerical chromosomes [13].

In this work, genome composition by array-CGH of a more diverse set of wine and brewing *S. cerevisiae* × *S. kudriavzevii* natural hybrids from diverse origins was evaluated to decipher their origins and evolution. The examination of gene losses and gains as well as the maintenance of specific metabolic pathways from the *S. cerevisiae* or *S. kudriavzevii* parental genomes was also analyzed with the aim of elucidating the role of each parental genome in the fermentative performance of the hybrid strains.

## Methods

### Yeast strains and culture media

The natural yeast hybrids *S. cerevisiae* × *S. kudriavzevii* used in this study have been isolated from wine and brewing fermentations in different locations (Table 1). The haploid strain *S. cerevisiae* S288c was used as control for microarray DNA hybridizations. Yeast strains were grown at 28°C in GPY medium (2% glucose, 0.5% peptone, 0.5% yeast extract).

**Table 1:** List of hybrid strains used in this study

**Strain name**	**Isolation source**
HA1841	wine, Perchtoldsdorf, Austria
HA1842	wine, Perchtoldsdorf, Austria
PB7	wine Pietro Picudo, León, Spain
Assmanhausen (AMH)	wine, Geisenheim, Germany
Anchor VIN7	commercial strain, Anchor, South Africa
SOY3	wine, Daruvar, Croacia
CECT1388	ale beer, United Kingdom
CECT1990	beer, Göttinger Brauhaus AG, Germany
CECT11002	beer Chimay Trappist, Belgium
CECT11003	beer Orval Trappist, Belgium
CECT11004	beer, Westmalle Trappist, Belgium
CECT11011	brewery, New Zealand

### Ploidy estimations by flow cytometry

Ploidy estimates are very important to interpret aCGH data from hybrids because hybridization signals are commonly normalized with respect to those of the reference haploid strain S288c.

The DNA content of both hybrid and control strains was assessed by flow cytometry by two different procedures. The first ploidy estimates were obtained in a FACScan cytometer (Becton Dickinson Inmunocytometry Systems, California, United States) by using the propidium iodide dye method described in Belloch et al. [13]. Due to discrepancies with the aCGH analysis, new estimates were later obtained in a Beckman Coulter FC 500 (Beckman Coulter Inc., California, USA) by using the SYTOX Green dye method described in Haase and Reed [14]. In both cases, ploidy levels were scored on the basis of the fluorescence intensity compared with the haploid (S288c) and diploid (FY1679) reference *S. cerevisiae* strains. Ploidy reported for each strain is the result of three independent measures. Results were tested by one way ANOVA and Tukey’s HSD tests.

### DNA labeling and microarray competitive genome hybridization

Total DNA, extracted as described in Querol et al. [15], was resuspended in 50 μl of de-ionized water and digested with endonuclease *Hinf* I (Roche Applied Science, Germany), according to the manufacturer’s instructions, to fragments of an average length of 0.25 to 8 kbp. Each sample was purified using High Pure PCR Product Purification Kit (Roche Applied Science, Germany) and 2 μg was labelled using BioPrime Array CGH Genomic Labelling System (Invitrogen, California, USA). Unincorporated label was removed using MinElute PCR Purification Kit (Qiagen, Germany). Equal amounts of labelled DNA from the corresponding hybrid strains and the control S288c strain were used as probes for microarray hybridization.

Array competitive genomic hybridization (aCGH) was performed using a double-spotted array containing 6,240 ORFs of *S. cerevisiae* plus control spots totaling 6.4 K (Microarray Centre, University Health Network, Toronto, Canada). New microarrays were pre-treated for one hour at 65°C with pre-hybridization solution (7.5 ml 20× SSC, 0.5 ml 10% SDS, 0.5 ml 10 mg/ml bovine serum albumin in 50 ml final volume). Pre-hybridization solution was washed during 15 s in mili-Q H_2_O, 2 s in 2-propanol, 2 s in milli-Q H_2_O and dried by centrifugation at 1200 rpm, 10 min. Microarrays were treated with hybridization solution (15 μl SSC, 0.6 μl 10% SDS, 6 μl 1 mg/ml salmon DNA and DNA labelled in 60 μl final volume) at 95°C for 1 min and at room temperature for 5 min before DNA hybridization. Hybridization was performed for 18 h in chamber at 65°C, thus allowing hybridization of the *S. cerevisiae* part of the hybrid genome. A negative control of microarray hybridization was done by using DNA from *S. kudriavzevii* IFO 1802 strain vs. S288c. After hybridization microarrays were washed at 65°C for 5 min in 2× SSC, 0.1% SDS, at room temperature in 0.1× SSC− 0.1% SDS for 10 min and six times in 0.1× SSC 1 min and dried by centrifugation at 1200 rpm, 10 min.

Experiments were carried out in duplicates and Cy5-dCTP and Cy3-dCTP dye-swap assays were performed to reduce dye-specific bias. The aCGH was performed for all hybrid strains except for W27, W46, SPG16-91 and SPG441 previously analyzed by Belloch et al. [13].

### Microarray scanning and data normalization

Microarray scanning was done by using a GenePix Personal 4100A scanner (Axon Instruments/Molecular Devices Corp., California, USA). Microarray images and raw data were produced with the GenePix Pro 6.1 software (Axon Instruments/Molecular Devices Corp., California, USA) and background was subtracted by applying the local feature background median option. M-A plots (M = Log_2_ ratios; A = log_2_ of the product of the intensities) were represented to evaluate if ratio data were intensity-dependent. The normalization process and filtering were done using Acuity 4.0 (Axon Instruments/Molecular Devices Corp., California, USA). Raw hybridization signals from hybrids were normalized with respect to those of the reference haploid strain S228c by using the ratio-based option, in which average hybridization ratios are adjusted to 1 (and hence, the corresponding log_2_ values to 0).

Normalized data were filtered by regression correlations 635/532 > 0.6, signal intensity in both channels more than 350 units, and signal to noise (SNR) > 2.5. Features with artifacts or flagged as bad were removed from the analysis. Replicates were averaged after filtering. It is worth to remark that strong normalization factors were applied to the negative control signal in each channel (2 to the red and 0.46 to the green one). Raw data and normalized microarray data are available in ArrayExpress [16], under the ref. E-MEXP-3114.

### Chromosome structure and recombination sites in the chimerical chromosomes

The log_2_ of normalized Cy5/Cy3 signal ratio obtained for each ORF was represented with respect to its corresponding chromosomal location using the completely sequenced reference *S. cerevisiae* strain S288c. These plots, called caryoscopes, were generated using ChARM v.1.1 [17]. Highly stringent hybridization conditions (65°C) were used to avoid the cross hybridization of *S. kudriavzevii* DNA present in the hybrids. The caryoscope of the negative control experiment showed that most *S. kudriavzevii* genes did not hybridize under these conditions and in the case of cross hybridization (red signal) this was due to the very strong normalization factors applied in these control, which increased the red signal and reduced the green one by factors not applied in the case of the experiments performed with DNA from hybrids (see Additional file 1: Figure S1). Accordingly, differences in the log_2_ ratio values observed in the caryoscopes revealed variations in the relative copy number of *S. cerevisiae* genes present in the hybrid strains.

The identification of over- and underrepresented regions was confirmed due to the normalization procedure, the hybridization ratios derived from aCGH analysis show the relative proportions of each gene with respect to the average number of copies in the hybrid, allowing the identification of over- and underrepresented regions in the hybrid genome by a one-way ANOVA test to determine the different levels of hybridization observed in the aCGH analysis. The approximate locations of the recombination points in the mosaic chromosomes were determined from the up and down jump locations in the ORFs mapping by microarray analysis of the hybrid yeast genomes.

Finally, by considering the collinearity of *S. kudriavzevii* and *S. cerevisiae* genomes [18], the S*. kudriavzevii* gene content in the hybrid genomes can be deduced from the presence/absence of the chromosome regions coming from each parental species, obtained in a previous PCR-RFLP analysis of these hybrids [19].

### Gene Ontology (GO) analysis of *S. kudriavzevii* genes

GenMAPP v2.1 software [20] was used to perform gene ontology analysis of the *S. kudriavzevii* fraction in the hybrid genomes. Four different GO analyses were carried out using *S. kudriavzevii* genes present in all hybrid strains, including those previously characterized [13], these analyses corresponded to: i) the complete set of wine and brewing hybrids, except strain AMH, showing the lowest *S. kudriavzevii* gene content, ii) only wine hybrids, except AMH, iii) only brewing hybrids and iv) only AMH. In all cases, statistically significant GO term enrichments were shown by computing a *p*-value using the hypergeometric distribution (the background set of genes was 6241, the number of ORFs measured in microarray experiments). GO terms showing significant values (z-score >2 and *p*-value <0.05) were sorted according to their corresponding GO category.

### Maximum parsimony tree

A list of minimal number of chromosomal rearrangements, chromosomal losses and restriction site changes were used to reconstruct the maximum parsimony tree. Data obtained from a previous study [13] were again included in this analysis. A binary matrix was constructed to codify each particular event (Additional file 2: Table S1). Parsimony trees were constructed by PHYLIP 3.66 package using the Mix program [21], taking chromosomal rearrangements and gain/losses as irreversible events (Camin-Sokal model) and the RFLP changes as reversible events (Wagner model). The consensus tree was obtained with Consense program using the Majority rule.

## Results

### Hybrid genome structures

Caryoscopes, representing log_2_ hybridization ratios for each gene mapped onto its corresponding chromosome position, of six hybrid strains from wine and 6 hybrids from brewing were obtained by array comparative genomic hybridization (aCGH) (Additional file 3: Figure S2). Due to the normalization procedure, the hybridization ratios derived from aCGH analysis show the relative proportions of each gene with respect to the average number of copies in the hybrid, allowing the identification of over- and underrepresented regions in the hybrid genome. However, aCGH analysis in combination with ploidy estimates and with information on the presence/absence of the chromosome regions coming from each parental species, obtained in a previous PCR-RFLP analysis of these hybrids [2,19], allowed us to decipher the genome composition of hybrids.

This way, ploidy estimates for these hybrids were obtained by flow cytometry. The initial estimates with the propidium iodide method suggested that most hybrids were diploids or close to diploidy (relative C-values of 2.0 to 2.6). However, these ploidy values were not congruent with the caryoscope and PCR-RFLP data. The ratio-based normalization of hybridization signals adjusts the average signal ratios (problem strain/reference strain) to 1, and hence the log_2_ values to 0. In the analysis of hybrids, ploidy estimates were 2n-2.6n, corresponding on average to a subgenome coming from each parental species, i.e. for each gene there are on average a copy coming from *S. cerevisiae* and another from *S. kudriavzevii*. Due to the high astringent hybridization conditions used in the aCGH analysis of hybrids, only the *S. cerevisiae* subgenome is hybridizing, as confirmed by the negative control performed with *S. kudriavzevii* DNA. Therefore, in the normalization of hybridization signals, these ratios correspond to the adjustment of average signals coming from 1 *S. cerevisiae* gene copy from the hybrid to 1 gene copy form the reference haploid *S. cerevisiae* strain. In the case of an increase of copy numbers in specific genes or chromosomal regions, log_2_ values should be higher than 0 (1, 2, etc. depending on the number of copies), but in the case of loss of *S. cerevisiae* gene copies in the hybrid, a ratio of 0 (log_2_ of – ∞) should be observed. However, 3–4 levels of log_2_ values, including negative but not infinite, are observed for some hybrids (Additional file 3: Figure S2), which made difficult the interpretation of the aCGH results and suggested that ploidy estimates with propidium iodide were wrong.

Therefore, new ploidy estimates of hybrids were obtained by using SYTOX Green as the DNA-binding dye, because Haase and Reed [14] demonstrated that improves linearity between DNA content and fluorescence, and decreases peak drift associated with changes in dye concentration, growth conditions or cell size. In this new ploidy analysis, Swiss wine hybrids analyzed in our previous study [13] were also included.

The statistical analysis of the new estimates showed two significantly different groups of hybrids according to ploidy levels: most hybrids, including the Swiss wine strains, appear as allotriploids and hybrids AMH and PB7 as allotetraploid yeasts (Table 2). The new ploidy estimates are in agreement with the different levels of hybridization observed in the aCGH analyses and also with the previous PCR-RFLP analysis of hybrids [19]. 

**Table 2:** **DNA contents of natural hybrids, estimated by flow cytometry using the SYTOX green method with respect to the reference haploid and diploid* S. cerevisiae* strains, S288c and FY 1679, respectively**

**Strain**	**DNA content relative to haploid strain S288c**
FY1679	2.00^a^ ± 0.00
HA 1841	3.01^b^ ± 0.08
HA 1842	3.07^b^ ± 0.07
VIN7	3.04^b^ ± 0.08
SOY3	2.89^b^ ± 0.09
CECT 1388	3.25^b^ ± 0.09
CECT 1990	2.86^b^ ± 0.07
CECT 11002	3.02^b^ ± 0.14
CECT 11003	3.21^b^ ± 0.09
CECT 11004	3.13^b^ ± 0.07
CECT 11011	2.99^b^ ± 0.05
W27	3.18^b^ ± 0.08
W46	3.20^b^ ± 0.07
441	3.10^b^ ± 0.09
SPG16-91	3.14^b^ ± 0.08
PB7	3.96^c^ ± 0.08
AMH	3.85^c^ ± 0.18

Results are the mean value of three replicates. Means with the same letters do not differ significantly by one way ANOVA and Tukey’s HSD tests (p < 0.05).

**Figure 1:**
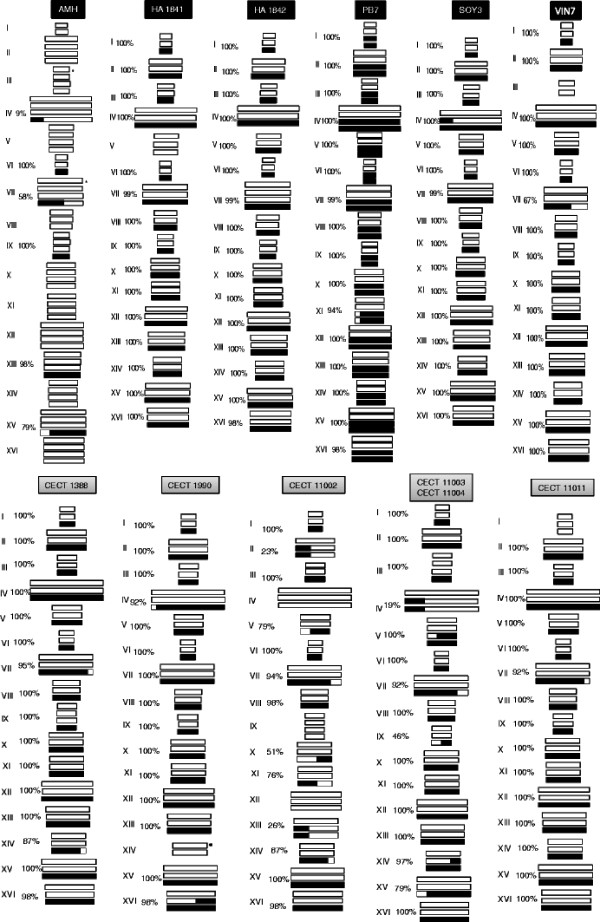
** Genome composition of hybrids deduced from aCGH analysis, ploidy estimates and a previous analysis of absence/presence of parental genes by RFLP analysis [**[2]**,**[19]**].** White and black bars are used to represent the *S. cerevisiae* and *S. kudriavzevii* genome fractions, respectively. Chromosomes showing black and white regions correspond to chimerical chromosomes. The percentages of *S. kudriavzevii* genes maintained in each chromosome are shown for each chormosome. Strains names are depicted on a black or a gray background corresponding to wine or brewing strains, respectively. Asterisks in AMH Chr. III and VII indicate regions where non-reciprocal translocations or segmental duplications can be present.

Final genome compositions were inferred for all hybrids as depicted in Figure 1, taking into consideration flow cytometry data, aCGH and PCR-RFLP data. For example, in the case of the partial allotetraploid hybrid AMH, its caryoscope shows four different hybridization levels, which correspond to 2 copies of *S. cerevisiae* genes located in chromosomes (chr.) I and VI; 3 copies of *S. cerevisiae* genes located in chr. VIII, IX and XIII, short chr. IV left region, chr. VII left arm, and chr. XV right arm; 4 copies of *S. cerevisiae* genes located in chr. II, III left, IV right, V, VII right, X, XI, XII, XIV, XV left, and XVI; and 5 copies of *S. cerevisiae* genes located in chr. III left region and in a segment of chr. VII (Figure 1; Additional file 3: Figure S2).

According to this combined analysis, 11 different patterns were differentiated in the 12 hybrids under analysis. As a general rule, different degrees of loss of *S. kudriavzevii* gene content in most hybrids were observed. Only the allotetraploid hybrid PB7 maintains a complete diploid set of chromosomes from each parental species, with the exception of a small segment located in the left arm of chromosome XI of the *S. kudriavzevii* subgenome. On the contrary, the largest reduction of the *S. kudriavzevii* gene content is observed in the partial allotetraploid hybrid AMH, which lost 72% of the *S. kudriavzevii* genes. The rest of hybrids, all of them allotriploid, showed intermediate situations derived from ancestors containing a diploid set of *S. cerevisiae* chromosomes and a haploid set of *S. kudriavzevii* chromosomes.

These combined analyses also allowed us to detect different types of chromosome rearrangements present in hybrids: i) the complete loss of a *S. kudriavzevii* parental chromosome compensated by an extra copy of the *S. cerevisiae* chromosome (chr. II,,III, V, X, XI, XII, XIV and XVI in AMH; chr. V in HA1841; chr. IV, IX and XII in CECT 11002; chr. I in CECT 11011); ii) aneuploidies (chr. I, VI and VIII in AMH; chr. IX in CECT 1388; chr. XIV in CECT 1990; chr. IX in CECT 11002; chr. III and V in CECT 11003 and CECT 11004; chr. III in VIN7), and iii) the presence of chimerical chromosomes (chr. IV, VII and XV in AMH; chr. XI in PB7; chr. IV in SOY3; chr. VII in VIN7; chr. VII and XIV in CECT 1388; chr. IV and XVI CECT 1990; chr. II, V, VII, X, XI, XIII and XIV in CECT 11002; chr. IV, V, VII, IX, XIV and XV in CECT 11003 and CECT 11004; and chr. VII in CECT 11011); (see Figure 1).

These chimerical chromosomes are characterized by over- and underrepresented regions evidenced as up and down jumps in the log_2_ ratio in the caryoscopes, which are indicative of probable non-reciprocal recombination events between homeologous chromosomes (homologous from different species) (Table 3). The recombination sites in the chimerical chromosomes were mapped according to the genome browser from Saccharomyces genome database (SGD). Using a windows size of 15–20 Kb (four genes in the left and right of the most plausible recombination point) we found Ty elements, ARS sequences, clusters of homologous regions (CHRs) and tRNA elements that may have facilitated the recombination of the two homologous parental chromosomes (Table 3). In several cases, a common recombination site was observed in chromosomes belonging to two or more hybrids, indicative of common ancestry. This is the case of chromosomes IV, V, IX, XIV and XV in brewing hybrids CECT 11003 and 11004; chromosome XIV in CECT 1388 and 11002; chromosome XV in CECT 11003, 11004 and AMH and chromosome VII in hybrids CECT 11003, CECT 11004, CECT 11002, CECT 11011 and CECT 1388 (Table 3 and Additional file 3: Figure S2).

**Table 3:** **List of chimerical chromosome (CC) types found in the different *S. cerevisiae*** × ***S. kudriavzevii* hybrids**

**Chr.**	**CC type**	**Strains**	**Breakpoint mapping interval**	**Putative recombining sequences**
II	type 1	**CECT 11002**	YBL018C-YBL011W	Ty1 LTR, Ty3 LTR, tRNA-Ile, tRNA-Gly, ARS
IV	type 1	*W27, W46, 441, SPG16-91*, **CECT 11003, CECT 11004**	YDL095W	*PMT1* (ref. [13])
	type 2	*AMH, SOY3*	YDL185W-YDL179W	CHR 12
	type 3	**CECT 1990**	YDL185W-YDL179W	CHR 12
V	type 1	*W27, W46, 441, SPG16-91*, **CECT 11003, CECT 11004**	YER006W	*NUG1* (ref. [13])
	type 2	**CECT 11002**	YEL018C-YEL011W	Ty1 LTR, Ty4 LTR, tRNA-Gln
VII	type 1	*W46,***CECT 11003, CECT 11004, CECT 11002, CECT 11011, CECT 1388**	YGR249W-YGR244C	ARS, CHR 29
	type 2	*AMH*	YGR062C-YGR058W	CHR 30
	type 3	*VIN7*	YGR106C-YGR112C	tRNA-Leu, tRNA-Lys, Ty1 LTR, tRNA-Cys, Ty3 LTR, ARS
IX	type 1	*W27, W46, 441, SPG16-91*, **CECT 11003, CECT 11004**	YIL053W	*RHR2-RPL34B* (ref. [13])
X	type 1	**CECT 11002**	YJL039C-YJL036C	tRNA-Asp, tRNA-Arg, Ty1 LTR, ARS, tRNA-Val
XI	type 1	**CECT 11002**	YKR025C-YKR028W	Ty1 LTR
	type 2	*PB7*	YKL203C-YKL204W	ARS
XIII	type 1	**CECT 11002**	YML012C-YML009W-B	CEN13, ARS
XIV	type 1	*W27, W46, 441, SPG16-91*, **CECT 11003, CECT 11004**	YNR001C	CEN14 (ref. [13])
	type 2	**CECT 1388, CECT 11002**	YNR029C-YNR032W	ARS
XV	type 1	*W27, W46, 441, SPG16-91***, CECT 11003, CECT 11004,***AMH*	YOL053W	*THI20-PSH1* (ref. [13])
XVI	type 1	**CECT 1990**	YPR007C-YPR011C	Ty1LTR, tRNA-Gly, tRNA-Lys,

Chr., chromosome number; CHR, cluster of homology region. Strain names in italics correspond to wine hybrids and in bold to brewing hybrids. Some recombination sites were described elsewhere [13], as indicated.

### *S. cerevisiae* gene depletions in hybrids

Although hybrids maintain in their genomes at least a complete set of *S. cerevisiae* chromosomes, aCGH data from all hybrids analyzed in this work, as well as from those previously analyzed [13], can be used to determine the common fraction of *S. cerevisiae* genes showing gene copy variations in hybrids compared to the reference strain S288c*.* A common set of genes showing the same copy number variations in hybrids may be indicative of common origins.

The analysis of the *S. cerevisiae* gene content from all hybrids revealed the presence of less copies of a common set of genes. Among them, the most interesting were *CUP1*, *ASP3,* and *ENA* gene families, as well as Ty elements and 13 ORFs of unknown function (Additional file 4: Table S2). In general, copy variations in the *S. cerevisiae* genome fraction of the hybrids were found in genes located in subtelomeric regions (Additional file 3: Figure S2), although in some cases involve genes located in intrachromosomal regions, such as *CUP1*.

Short segment amplifications were also detected in the aCGH analysis. This was the case of hybrid AMH that showed three short region amplifications in chr. III, VII and XIII. The higher hybridization signals of genes located in the two first regions could be postulated as indicative of the presence of chimerical chromosomes, however according to the previous PCR-RFLP analysis *S. kudriavzevii* genes were absent. Other amplifications of *S. cerevisiae* segments located in chromosome XVI are observed in hybrids CECT 1388 (between genes YPL159C and YPL126W) and CECT 11002 (between YPL141C and YPL126W). Finally, a deleted region was found in one of the two copies of *S. cerevisiae* chromosome XIV from strain CECT 1990 (between loci YNR013C and YNR031C) (Additional file 3: Figure S2).

### *S. kudriavzevii* gene content and Gene Ontology (GO) analyses

Data obtained from all hybrids analyzed in this work as well as from those previously analyzed [13] were also used to evaluate the presence of common *S. kudriavzevii* genes (Additional file 5: Table S3). These common set of genes could be interesting to unveil potentially genes of adaptive value in hybrids.

As a general rule, most hybrids maintained around 90% of the *S. kudriavzevii* genome, with the exception of the brewing strain CECT 11002 and the wine strain AMH which only maintain 56.9% and 30.5% respectively.

To determine if a group of *S. kudriavzevii* genes associated with particular cellular components, molecular functions or biological processes may have been maintained in all hybrids due to potential adaptive value, four different gene ontology (GO) term enrichment analyses were performed (Additional file 6: Table S4). The first analysis included all wine and brewing hybrids. Due to the low representation of the *S. kudriavzevii* genome fraction in AMH, this strain was removed from this first analysis. Gene ontology analysis was also separately performed according to the source of isolation of hybrids, wine and brewing fermentations. GO terms showing significant values were sorted according to their corresponding GO categories (Additional file 6: Table S4). Table 4 shows only those significantly represented GO terms of putative importance for wine or brewing fermentations.

**Table 4:** **Summary of the most relevant metabolic pathways and biological processes obtained after Gene Ontology analysis using the* S. kudriavzevii* genes retained in each group of hybrids**

**Group of hybrids**	**GO ID**	**GO Term**	**N**_**present**_**/N**_**measured**_	**%**	**p-value**
**WINE**	6487	Protein amino acid N-linked glycosylation	36/42	85.7	0.013
	6839	Mitochondrial transport	10/10	100	0.033
		Ergosterol Biosynthesis	17/19	89.5	0.049
**BREWING**	6487	Protein amino acid N-linked glycosylation	28/42	66.7	0.017
		Fatty acid elongation saturated	4/4	100	0.039
		Glycine serine and threonine metabolism	27/42	64.3	0.03
		Arginine_and_proline_metabolism	16/23	69.6	0.049
		Sulfur_Degradation	4/4	100	0.048
**ALL**	6487	Protein amino acid N-linked glycosylation	25/42	59.5	0.003
	15908	Fatty acid transport	4/4	100	0.025
		Glutamate metabolism	15/27	55.6	0.046
		Sulfur metabolism	8/11	72.7	0.021
		NAD salvage pathway	5/6	83.3	0.027
		Sulfate assimilation pathway II	5/6	83.3	0.019
**AMH**	6972	Hyperosmotic response	5/7	71.4	0.036
	9331	Glycerol 3 phosphate dehydrogenase complex	3/3	100	0.033
		Histidine biosynthesis	5/7	71.4	0.039
		Fatty acid metabolism	11/17	64.7	0.010

Due to the massive S. kudriavzevii gene losses in AMH, this strain was not included in any grouping, and hence, analyzed alone.

Significantly represented GO terms common to both wine and brewing hybrids mainly corresponded to genes related to fatty acid metabolism (particularly transport), sulfur metabolism and the NAD^+^ salvage pathway. Genes associated with amino acid metabolism (N-linked glycosylation and glutamate metabolism) were also represented (Table 4).

GO terms related to amino acid N-linked glycosilation were also significantly present in hybrids from wine and brewing analyzed independently. Moreover, GO terms associated with ergosterol biosynthesis and mitochondrial transport were also significantly detected in wine hybrids; while those related to metabolism of amino acids such as glycine, threonine, arginine and proline, sulfur metabolism, as well as fatty acid elongation were significant present in brewing strains (Table 4). Finally, an independent analysis of significant GO terms for AMH hybrid revealed the presence of genes involved in hyperosmotic response, glycerol-3-phosphate dehydrogenase complex, histidine biosynthesis and fatty acid metabolism (Table 4).

### Phylogenetic relationships among hybrids

**Figure 2:**
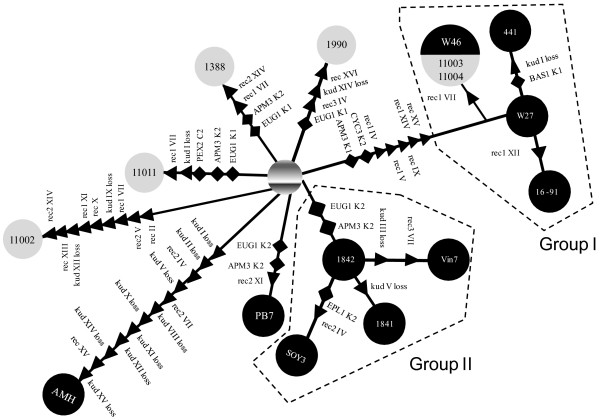
** Maximum parsimony tree indicating the minimum number of chromosomal rearrangements and restriction site changes (presence/absence matrix is given in Additional file**2**: Table S1) necessary to connect the different genotypes exhibited by the *S. cerevisiae *×* S. kudriavzevii* hybrids to a putative hybrid ancestor.** This putative ancestor is not necessarily the same for all lineages, it just corresponds to an ancestral state containing the complete *S. cerevisiae* and *S. kudriavzevii* genomes, but it could be generated several times from different parental strains, as discussed in the main text. Genotypes are represented by white and gray circles for wine and brewing hybrids, respectively. Rearrangements are indicated by arrows giving the direction of the irreversible change and were treated under the Camin-Sokal criterion. Rearrangements were assumed to be caused by nonreciprocal recombination (rec) among homoeologous chromosomes (roman numbers) and whole chromosome losses (loss) of one of the parental chromosomes (*kud*, *S. kudriavzevii*). Restriction site changes can be reversible (gains/losses represented by diamonds) and were treated under the Wagner criterion. The gene region and the restriction patterns involved are also indicated (for a description see references [2] and [19]).

A maximum parsimony tree was constructed based in presence/absence of chromosomes and chromosome regions data obtained for each particular genetic event in all analyzed hybrids. The tree topology revealed the presence of two main groups containing most allotriploid hybrids, particularly those from wine (Figure 2).

Group I was constituted by Swiss wine strains W46, 441, W27 and SPG 16-91 as well as the brewing strains CECT 11003 and CECT 11004. This group is supported by the presence of five shared chimerical chromosomes as well as the *CYC3* K2 allele [2].

Group II includes the remaining allotriploid wine hybrids HA1841, HA 1842, VIN7 and SOY3. This group is only supported by the common presence of *S. kudriavzevii* K2 alleles for genes *EUG1* and *APM3*[19], and the possession of a higher fraction of *S. kudriavzevii* genome*.*

The rest of the allotriploid hybrids, isolated from brewing, and the wine allotetraploid PB7 and AMH strains, appeared in separated branches with strain-specific chromosomal rearrangements. The only exception is the shared loss of *S. kudriavzevii* chr. XII between the partial allotetraploid AMH and the allotriploid CECT 11002, which can be considered a convergent event. PB7 also shared similar restriction alleles with Group II but this strain is also allotetraploid (Table 2).

This most parsimonious tree shows several convergent events, such as chromosomal losses, chromosomal rearrangements and restriction site changes (evidencing different allelic variants). *S. kudriavzevii* chr. I seems to have been lost independently in hybrids SPG 441, CECT 11011, and AMH. In a similar way, the lack of chr. V in hybrids HA 1841 and AMH, and chromosome XIV in CECT 1990 and AMH seem to be independent events according to this parsimony analysis.

Convergent events involving recombinant chromosomes were also found. This is the case of the type 2 recombination in chr. IV (shared by AMH and SOY3), type 1 recombination in chr. VII (shared by CECT 11002, CECT 11011, 1388, W46, CECT 11003 and CECT 11004), type 2 recombination in chr. XIV (CECT 1388 and CECT 11002) and the recombinant chr. XV (AMH and Group I hybrids). This could be indicative of the presence of recombination hotspots in the *Saccharomyces* genomes.

## Discussion

### The genome diversity in *S. cerevisiae* × *S. kudriavzevii* hybrids

The genome composition of 11 new wine and brewing *S. cerevisiae* x *S. kudriavzevii* hybrid strains was described in this work by means of aCGH analysis. Additionally, a comparison between them and other four wine hybrids already described by [13] was also performed. Individual and differential chromosomal composition patterns were found for each particular strain, except for brewing strains CECT 11003 and CECT 11004 which appear closely related to the previously described Swiss wine hybrids [13]. The close relationships between wine hybrid strains from Switzerland and the brewing strains CECT 11003 and 11004 was already observed in a previous study based on PCR-RFLP analysis of hybrids as well as in the phylogenetic reconstruction based on COX2 sequences [19]. In that work, a recombination in chromosome XV was proposed as the unique difference between strains 11003 and 11004; however, aCGH analysis carried out in this study demonstrated that this recombination is present in both strains (Figure 2). These Swiss wine hybrids were previously described as diploids [13] on the basis of ploidy estimations with propidium iodide. However, in the reanalysis of ploidy with SYTOX Green, they also resulted to be allotriploids as CECT 11003 and CECT 11004.

Flow cytometry results with SYTOX Green were in accordance with genome structure deduced from aCGH analysis carried out in this work and with the presence/absence of parental genes deduced from a previous PCR-RFLP analysis of hybrids [19]. Most *S. cerevisiae* × *S. kudriavzevii* hybrid strains were allotriploids, with the exception of AMH and PB7 which were allotetraploids. Some aneuploidies were also found in several hybrids. Aneuploidies seem to be common in *Saccharomyces* hybrids since this phenomenon have also been observed in *S. cerevisiae* × *S. bayanus* hybrids [22,23]. The role of aneuploidies in the hybrid genomes is not clear, but their presence in *S. cerevisiae* affected both the transcriptome and proteome, generating significant phenotypic variation and bringing fitness gains under diverse conditions [24].

Recently, the hybrid genome of VIN7, one the hybrids analyzed in the present study, has completely been sequenced [25], concluding that this strain is an almost perfect allotriploid hybrid that contains a heterozygous diploid *S. cerevisiae* genome and a haploid *S. kudriavzevii* genome. The genome constitution of VIN7 deduced from the sequencing analysis is basically similar to the one inferred by aCGH in the present study, but there are some differences. The genome sequence analysis detected a homeologous recombination generating a chimerical chromosome VII, a genomic substitution of a region of 15 kb, of *S. kudriavzevii* genomic DNA from chromosome IV by the orthologous sequences from *S. cerevisiae* and a genomic substitution of a 13 kb region of *S. cerevisiae* genomic DNA from chromosome IV by *S. kudriavzevii* sequences combined with homeologous recombination between the *S. kudriavzevii* and *S. cerevisiae* alleles. The first rearrangement involving a chimerical chromosome VII was clearly detected in the aCGH analysis, but not the two genomic substitutions. Both genomic substitution involve short segmental replacements of a few genes (7 and 8), and the second an almost reciprocal recombination between homeologous chromosomes that cannot be observed by aCGH analysis. However, the presence/absence analysis of parental genes in hybrids [19] detected the loss of *S. kudriavzevii* chromosome III in our VIN7. As an ongoing project, our group is also sequencing the whole genome of several *S. cerevisae* × *S. kudriavzevii* hybrids, including the commercial VIN7 yeast. We checked in the preliminary sequencing of our VIN7 strain for the presence of *S. kudriavzevii* chromosome III sequences and the result was negative, confirming our aCGH results and indicating that our VIN7 strain is different. These differences may be due to the fact that our VIN7 strain was isolated from a commercial dry yeast sample provided by Anchor Yeast but Borneman et al. [25] sequenced the original mother culture of VIN7, as they mention in their acknowledgements. Therefore, the continuous propagation of this yeast in molasses under aerobic conditions to obtain commercial dry yeasts may have promoted a new chromosomal rearrangement, the loss of the *S. kudriavzevii* chromosome III.

Taking into consideration the ploidy data as well the fact that most hybrids possess either trisomic (2 *S. cerevisiae* chromosomes: 1 *S. kudriavzevii* chromosome) or tetrasomic chromosomes (2 *S. cerevisiae* chromosomes: 2 *S.kudriavzevii* chromosomes), two scenarios on the hybridization process are plausible. In the case of allotriploid hybrids, the simplest explanations for their origins are hybridization events by rare-mating between a diploid cell of *S. cerevisiae* and a haploid cell or spore of *S. kudriavzevii*. This is also supported by the genome sequencing of VIN7, one of the allotriploid strains, which resulted to contain heterozygous diploid genome from *S. cerevisiae* and a haploid genome from *S. kudriavzevii*[25].

On the other hand, diploid and diploid cell rare-mating between *S. cerevisiae* and *S. kudriavzevii* should be invoked to explain the origin of allotetraploid hybrids. In the case of PB7 it was observed high spore viability (95%) due to the presence of the two chromosomes copies of each parental strain.

Rare-mating between diploid cells was already proposed as a probable mechanism for hybrids generation [13,26]. However, haploid cell or spore mating between *S. cerevisiae* and *S. kudriavzevii*, followed by whole genome duplications due to endoreplication or chromosome duplications due to non-disjunction, and subsequent chromosomal rearrangements, although less plausible, cannot totally be discarded.

### Characterization of the *S. kudriavzevii* subgenome from hybrids

According to Sipiczki [26], genomes from each parental species interact in the new hybrid genome. This interaction can be observed in the loss of large parts of one or both genomes as well as in the presence of chimerical chromosomes that make the hybrid genome as stable as possible to future genetic modifications. Additionally, adaptive evolution of these hybrid genomes under fermentative environmental conditions could make hybrid genome to conserve the chromosomes, or part of them, which grant a selective advantage [27]. According to the results obtained in this work as well as in our previous studies [2,13,19], *S. cerevisiae* × *S. kudriavzevii* hybrids seem to have the common trend to lose the *S. kudriavevii* parental chromosomes maintaining the *S. cerevisiae* ones. The reduction of the non-*S. cerevisiae* genome observed in both wine and brewing *S. cerevisiae* × *S. kudriavzevii* hybrids was already reported for artificial *S. cerevisiae* × *S. uvarum* hybrids genetically stabilized by successive sporulation steps [28]. In contrast, *S. pastorianus* (*S. cerevisiae* × *S. eubayanus* hybrids) Group 1 strains obtained from different brewing processes and studied by aCGH analysis, showed a trend to lose the *S. cerevisiae* genome fraction [22]. The cause of the predominance of one or the other parental genome in the hybrids remains unclear yet. However, selective pressures acting under harsh environmental conditions and cytonuclear interactions have been suggested as the main factors affecting the genome conformation of hybrids. In *S. cerevisiae* × *S. eubayanus* lager strains, supposed to be naturally selected after years of use in brewing, the predominance of a *S. eubayanus-*like genome has been related to the maintenance of the *S. eubayanus* mitochondria [22,29]. However, artificial hybrids constructed from the same two parental species, but without selective pressures, inherited their mitochondrial genome from either one or the other parental species randomly [29,30]. The conservation of the mitochondrial genome from the parental species most represented in the nuclear genome was also observed in the stable artificial *S. cerevisiae* × *S. uvarum* hybrids, which maintained the mitochondrial genome of the *S. cerevisiae* parental strain [28]. All *S. cerevisiae* × *S. kudriavzevii* natural hybrids analyzed in this work, except for AMH, maintained a *S. kudriavzevii* mitochondrial genome [2,19]. However, *S. cerevisiae* × *S. kudriavzevii* artificial hybrids, randomly inherited the *S. cerevisiae* or the *S. kudriavzevii* mitochondrial DNA (Pérez-Través et al. personal communication). This discrepancy between the mtDNA inheritance in artificial vs. natural hybrids has been associated with the result of an unwitting human-driven selection of naturally generated hybrid strains for fermentations at low temperature [29]. A common origin for all hybrids could be another possible explanation, but the present analysis of the genome constitutions in hybrids suggests diverse origins.

Interestingly, the hybrid AMH, which maintained the *S. cerevisiae* mitochondria, has lost a 69% of the nuclear genes of *S. kudriavzevii* coding for proteins with functions associated to the mitochondria; while the rest of the analyzed hybrids with *S. kudriavzevii* mitochondria have lost only 0.67%–42.48% of the *S. kudriavzevii* genes related to mitochondrial functions. Due to the fact that a number of mitochondrial proteins encoded in the nuclear genome play an important role in the mtDNA replication and transmission, both the type of mitochondrial DNAs and the functions of the mitochondria in a hybrid strain are clearly under the control of the nuclear genome [31]. One of the most interesting evidence about nuclear-mitochondrial genome interactions were described by Lee et al. [32], who demonstrated that the presence of the *S. bayanus* nuclear gene *AEP2* together with the *S. cerevisiae* mitochondrial gene *OLI1* cause a cytonuclear incompatibility. More recently, Chou et al. [33] identified other two genes, *MRS1* and *AIM22*, associated with cytonuclear incompatibility among *S. cerevisiae*, *S. paradoxus* and *S. bayanus*. A similar behavior involving the same or other different genes in *S. cerevisiae* × *S. kudriavzevii* hybrids was not yet demonstrated.

aCGH and GO analysis carried out with those *S. kudriavzevii* genes conserved in all *S. cerevisiae* × *S. kudriavzevii* hybrids with *S. kudriavzevii* mitochondria (excluding AMH) evidenced a significant enrichment in nuclear genes related to mitochondrial function (a total of 328 genes) supporting the hypothesis of a necessary interaction between the *S. kudriavzevii* nuclear-encoded proteins and the mitochondrial genomes or their products. Taking into consideration that a total of 751 proteins encoded by the nuclear genome are associated with the mitochondrial function in *S. cerevisiae*[34], and considering a similar number in *S. kudriavzevii*, we can assume that the remaining genes up to 751 might be non-essential for the maintenance of the *S. kudriavzevii* mitochondria in hybrids. In particular the *S. kudriavzevii* gene *AEP2* reported by Lee et al. [32] was not common to all analyzed hybrids, indicating that different incompatible nuclear-mitochondrial pair of genes could be associated with each particular pair of *Saccharomyces* parental species involved in hybrid generation.

GO analysis was also very informative with regards to the conservation in hybrids of particular groups of genes, inherited from each parental species, that may be potentially related to adaptive advantage for fermentation at low temperatures. A significant overrepresentation of *S. kudriavzevii* genes associated with the physiological adaptation of yeasts to grow at low temperatures, such as fatty acid transport and N-glycosilation of proteins in all hybrids, and ergosterol biosynthesis in the case of wine hybrids [35-37] was observed (Table 4). Changes in membrane fluidity are the primary signal triggering the cold shock response [35]. This response involves certain groups of genes: members of the *DAN*/*TIR* family of cell-wall mannoproteins, genes coding for temperature inducible protein (*TIP1*) and seripauperins (*PAU*), genes related to ergosterol and phospholipid synthesis (*ERG*, *INO1* and *OPI3*) and the gene coding for the only known desaturase in *S. cerevisiae* (*OLE1*), among others [35]. These sets of genes are present in the *S. kudriavzevii* subgenome of all hybrids analyzed in this work, with some exceptions mainly involving AMH (Table 4 and Additional file 6: Table S4).

Our results are in agreement with results about stress olerance, including adaptation to low temperatures, previously obtained in our laboratory using some of the *S. cerevisiae* × *S. kudriavzevii* hybrids analyzed in this work [11,38]. Physiological implications of possessing *S. kudriavzevii* genes in those particular functions or metabolic pathways must be elucidated in future studies involving both transcriptomic and metabolomic analyses.

### Wine yeast signatures in the *S. cerevisiae* subgenome from hybrids

An interesting result obtained from aCGH analysis was the detection of a common set of *S. cerevisiae* genes that are in lower copies in the genome of all *S. cerevisiae* × *S. kudriavzevii* hybrids (Additional file 4: Table S2). This finding might indicate that the *S. cerevisiae* parental strains involved in the different hybridization events shared a similar genetic background and were closely related yeasts.

Using a similar methodology, a trend to loss some particular set of genes in *S. cerevisiae* wine strains, with regards to strains belonging to the same species but isolated from different sources, was previously demonstrated [39,40]. Dunn et al. [39] proposed the term “commercial wine yeast signature” to refer to this set of genes. Most of these genes that are frequently depleted in wine strains are also depleted in the *S. cerevisiae* fraction of the hybrid genomes of all hybrids. This finding supports the hypothesis that these hybrids have likely been generated from wine *S. cerevisiae* parental strains.

### On the origin of hybrids

The maximum parsimony analysis of the relationships between the wine and beer hybrids are congruent with diverse origins for the strains according to chromosomal rearrangement differences, mainly due to the presence of chimerical chromosomes, and *S. kudriavzevii* chromosome losses, in some cases compensated by the presence of an extra copy of the homeologous *S. cerevisiae* chromosome (Figure 2).

While the brewing strains seem to represent different and divergent lines (except strains CECT11003 and 11004), most wine hybrids clustered in two main groups of strains sharing common events, with the exception of AMH and PB7 that were independently originated. Brewing strains CECT 11003 and 11004 shared the same genome than wine hybrid W46 probably evidencing that either an original strain with this common genome structure was introduced in both fermentative processes, or colonize one fermentative process from the other. The parsimony tree obtained in this study is congruent with previous phylogenetic reconstructions of hybrids based on COX2 sequences [19].

The occurrence of several chimerical chromosomes sharing similar—if not the same—recombination points, common to some *S. cerevisiae* × *S. kudriavzevii* hybrids located in different branches of the parsimony tree, indicates the presence of recombination hot spots. Recombination between homeologous chromosomes are probably mediated by highly recombining regions located in the recombination sites, such as ARS sequences [41], Ty elements [42], Y’ elements, rRNA regions and conserved coding genes [13,43]. When recombination is initiated in a region with high homology, the mismatch repair system (MMR) stimulates the loss of one partner of the recombination event in the hybrids and the fixation of the other, thus generating a chimerical recombinant chromosome. With the exception of the almost perfect allotetraploid PB7, hybrids have low spore viability (<1%) indicating that they are maintained by mitotic budding. Therefore, mitotic homeologous recombination, although much less frequent than meiotic, may also explain the generation of chimerical chromosomes.

The genome composition of hybrids reveals that the ancestral hybrid strains were allotriploid or allotetraploid, resulting from rare mating between diploid *S. cerevisiae* and haploid or diploid *S. kudriavzevii*[4,25]. The presence of triple hybrids also supports this hypothesis [1,19]. Finally, the presence of *S. kudriavzevii* alleles shared between most hybrids and the European *S. kudriavzevii* population [10], as well as the presence of the gene *GAL4* from *S. kudriavzevii*[2,19], which is a functional gene in the European populations of *S. kudriavzevii* but a pseudogene in the Japanese strains [44], indicate that these hybrids were originated from a European *S. kudriavzevii* parental strain.

## Conclusions

Hybridization between *S. cerevisiae* and *S. kudriavzevii* have occurred several times by rare-mating between different wine *S. cerevisiae* diploid and European *S. kudriavzevii* haploid or diploid progenitors. After hybridization, the hybrid genome suffered random genomic rearrangements mediated by crossing-over between homologous chromosomes and non-disjunction, promoting the loss of variable fractions of the parental subgenomes. Both the restrictions imposed byinteractions between both parental genomes as well as between nuclear and mitochondrial genomes, together with the selective environmental conditions prevailing during fermentation modulated the final composition of the hybrid genomes, characterized by the maintaining of the *S. cerevisiae* genome and the progressive reduction of the *S. kudriavzevii* contribution.

## Competing interests

The author(s) declare that they have no competing interests.

## Authors’ contributions

This study is the result of the collaboration between AQ and EB laboratories. CB, AQ and EB conceived and supervised this study. DP, CB, AQ and EB designed the experiments. DP and CL performed the experimental work and data analyses. DP and CB wrote the first version of the manuscript. CL, AQ and EB participated in the final manuscript revision. All authors read and approved the final manuscript.

## Supplementary Material

Additional file 1 ** Figure S1.** Caryoscope representation of microarray data of S.kudriavzevii IFO1802. Array CGH data are shown in numerical order with chromosome I at the top and chromosome XVI at the bottom. Red signal indicates hybridization signal but it’s important to note the high normalization factor applied to the red signal (2) and the correction applied to the green one (0.49). This figure indicate that no cross hybridization has occurred between *S. kudriavzevii* genes and *S. cerevisiae* genes. (PPTX 106 kb) Click here for file

Additional file 2 ** able S1.** Binary table showing the presence/absence of a particular event for each hybrid strain (coded as 1/0 respectively). kud, *S. kudriavzevii*; loss, chromosome loss; rec, recombination generating a chimerical chromosome. Roman numerals indicate chromosome numbers and Arabic numerals the types of chimerical chromosomes or the restriction sites in the analysis of RFLP patterns in 34 genes. Parsimony tree was computed using a mixture model in which chimerical chromosomes, *S. kudriavzevii* chromosome losses and double *S. cerevisiae* chromosome were considered under the Camin-Sokal criterion and restriction site gains/losses under the Wagner criterion. (XLS 32 kb) Click here for file

Additional file 3 ** Figure S2.** Caryoscope representation of microarray data of 11 *S. cerevisiae* × *S.kudriavzevii* hybrids. Array CGH data are shown in numerical order with chromosome I at the top and chromosome XVI at the bottom for each strain. Regions with higher red signal correspond to *S. cerevisiae* genome regions that are overrepresented in the hybrid genome and those with higher green signal to those regions that are underrepresented. aCGH of wine and brewery hybrids are depicted on black and gray backgrounds, respectively. Arrows indicate potential non-reciprocal recombination events between homeologous chromosomes involved in the generation of chimerical chromosomes. (PPT 607 kb) Click here for file

Additional file 4 ** Table S2.** List of *S. cerevisiae* genes depleted in all *S. cerevisiae* × *S. kudriavzevii* hybrids under analysis. (XLS 29 kb) Click here for file

Additional file 5 ** Table S3. ***S. kudriavzevii* gene composition for each hybrid. A cross indicates the putative presence of that gene by considering colinearity between *S. kudriavzevii* and *S. cerevisiae* genomes. R indicates interspecies recombinant genes. Wine and brewing hybrids are indicated in black and gray lettering, respectively. (XLS 2001 kb) Click here for file

Additional file 6 ** Table S4.** Metabolic pathways and biological processes obtained from a Gene Ontology analysis using the *S. kudriavzevii* genes retained in each hybrid grouping. (XLS 38 kb) Click here for file
