# Supplementary material for: Comparative genomics among Saccharomyces cerevisiae × Saccharomyces kudriavzevii natural hybrid strains isolated from wine and beer reveals different origins
Source: BMC Genomics. 2012 Aug 20;13:407. doi: 10.1186/1471-2164-13-407 (PMC3468397; doi:10.1186/1471-2164-13-407)
Supplement: Additional file 1 — Figure S1. Caryoscope representation of microarray data of S.kudriavzevii IFO1802. Array CGH data are shown in numerical order with chromosome I at the top and chromosome XVI at the bottom. Red signal indicates hybridization signal but it’s important to note the high normalization factor applied to the red signal (2) and the correction applied to the green one (0.49). This figure indicate that no cross hybridization has occurred between S. kudriavzevii genes and S. cerevisiae genes. (PPTX 106 kb) [file 1471-2164-13-407-S1.ppt]

## Slide 1
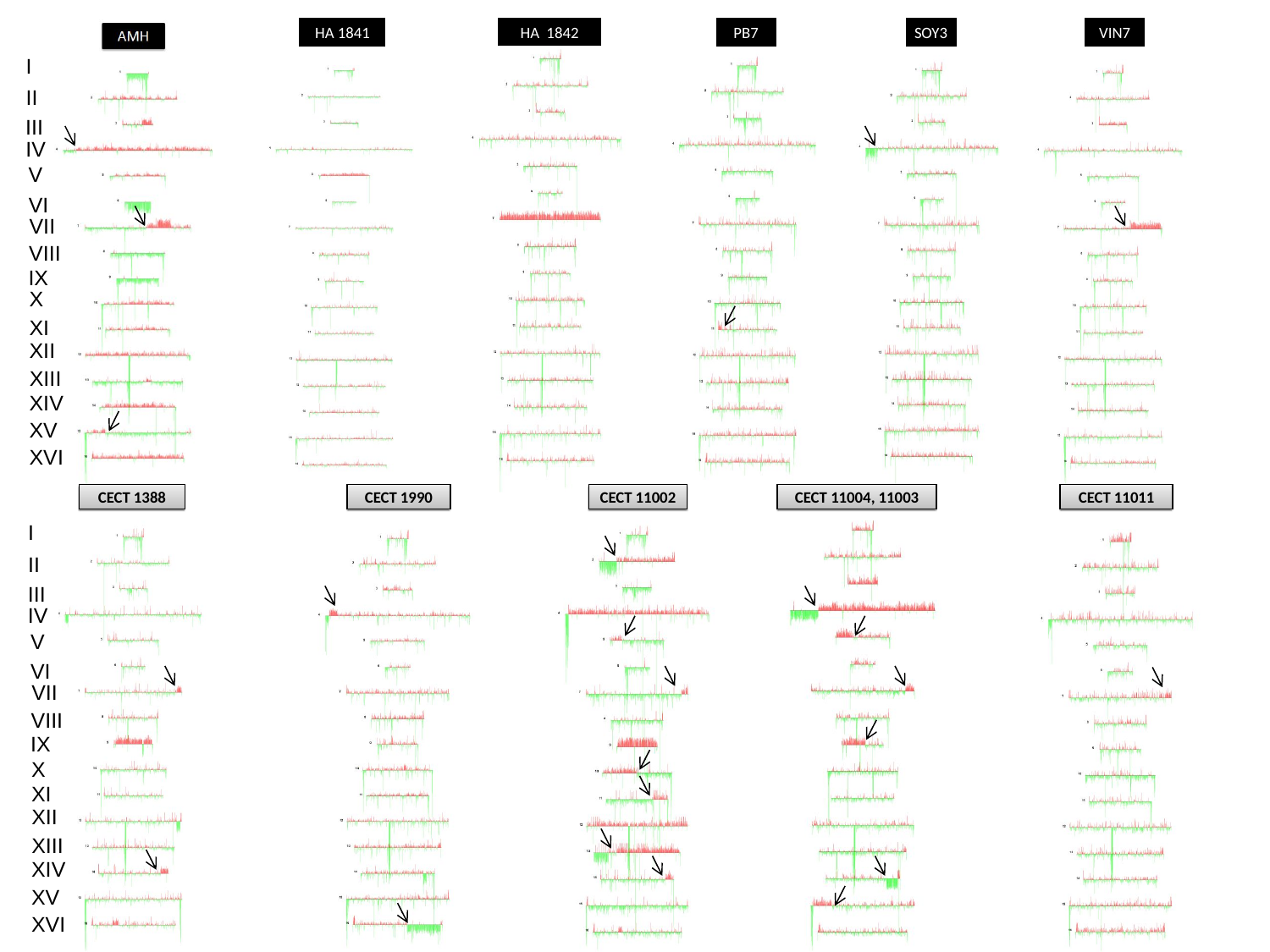

HA 1841
HA 1842
PB7
SOY3
VIN7
I
II
III
IV
V
VI
VII
VIII
IX
X
XI
XII
XIII
XIV
XV
XVI
CECT 1388
CECT 1990
CECT 11002
CECT 11004, 11003
CECT 11011
I
II
III
IV
V
VI
VII
VIII
IX
X
XI
XII
XIII
XIV
XV
XVI
