# Supplementary figures and images for: Comparative genomics among Saccharomyces cerevisiae × Saccharomyces kudriavzevii natural hybrid strains isolated from wine and beer reveals different origins
Source: BMC Genomics. 2012 Aug 20;13:407. doi: 10.1186/1471-2164-13-407 (PMC3468397; doi:10.1186/1471-2164-13-407)

## Slide 1
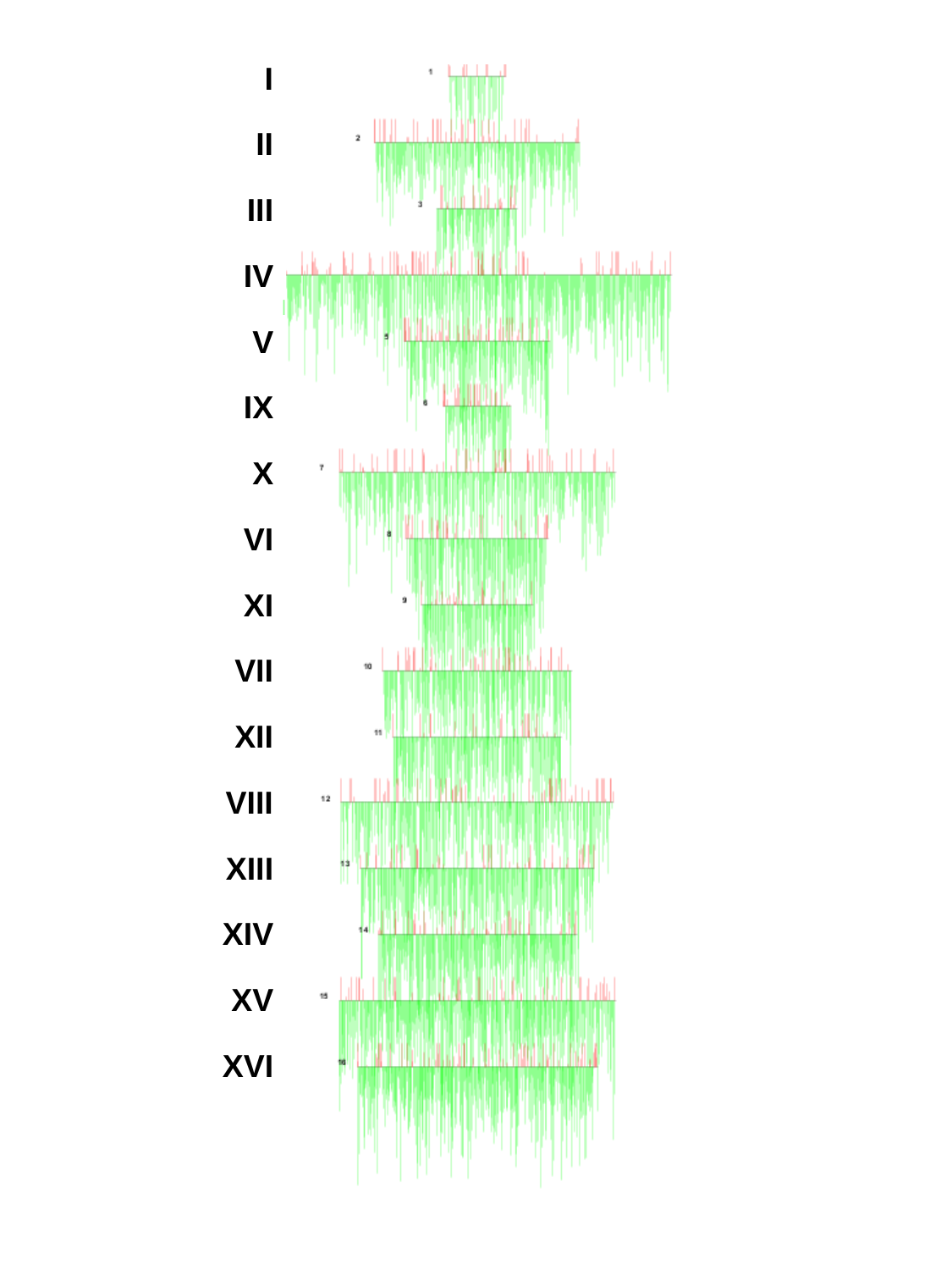

I
II
III
IV
V
IX
X
VI
XI
VII
XII
VIII
XIII
XIV
XV
XVI

Supplement: Additional file 6 — Table S4. Metabolic pathways and biological processes obtained from a Gene Ontology analysis using the S. kudriavzevii genes retained in each hybrid grouping. (XLS 38 kb) [file 1471-2164-13-407-S6.pptx]
